# Sexual Function after Partial Penectomy: A Prospectively Study From China

**DOI:** 10.1038/srep21862

**Published:** 2016-02-23

**Authors:** Cui Yu, Chen Hequn, Liu Longfei, Chen Minfeng, Chen Zhi, Zeng Feng, Chen Jinbo, Qi Lin, Zu Xiongbing

**Affiliations:** 1 Department of Urology, Xiangya Hospital, Central South University, Changsha, Hunan 410000, China

## Abstract

The Purpose of this study was to evaluate the sexual function after partial penectomy for penile carcinoma patients. Between January 2010 and May 2013, patients treated with partial penectomy at our institution were prospectively enrolled in this study. Sexual function (IIEF-15), age, body mass index, penile length in the flaccid state after partial penectomy (PL), treatment, having a partner and psychological factors (SAS scores and SDS scores) were assessed. Univariate and multivariate linear regression analyses were performed. 43 patients were included in our study. The median age was 56 years, and the median PL was 4 cm. The preoperative IIEF-15, SAS, SDS scores were significantly different from the postoperative scores. There was no statistically significant difference between the patients treated with partial penectomy and partial penectomy+ lymphadenectomy on IIEF-15 scores. Age was negatively associated with erectile function, sexual desire, and overall satisfaction; PL was positively associated with intercourse satisfaction; SAS score was negatively associated with erectile function, orgasmic function, sexual desire, and intercourse satisfaction. Our preliminary findings suggest that the sexual function after partial penectomy was significantly reduced. The sexual function was negatively affected by age and anxiety but positively affected by PL.

Penile carcinoma is a rare malignant disease with a significantly higher incidence in some developing countries. Partial penectomy is a common treatment for penile carcinoma. After partial penectomy, vaginal penetration is frequently possible. According to Romero’s study, 55.6% of patients reported erection of the penile allowed vaginal penetration after partial penectomy^1^. The sexual function of men with partial penectomy is often affected by physiological and psychological factors. However, few studies have provided information about the sexual function and the influenced factors after partial penectomy. The objective of this prospective study was to evaluate the sexual function after partial penectomy, and the associations between sexual function and age, body mass index (BMI), penile length in the flaccid state after partial penectomy (PL), treatment, partner and psychological factors.

## Materials and Methods

Between January 2010 and May 2013, penile carcinoma patients at our institution were prospectively enrolled in this study. The patients met the following selection criteria were eligible for study: 1) patients with penile carcinoma require partial penectomy. 2) Patients with regular sexual activity before surgery. Patients were excluded from our study if they had been treated with total penectomy, been treated with radiotherapy or chemotherapy, developed recurrent or metastasis disease. Laparoscopic bilateral inguinal lymphadenectomy (LBIL) was performed for patients with T2, T3 or T1G3 disease. These patients underwent LBIL within 3 months after partial penectomy. The methods were carried out in accordance with approved guidelines. The study was approved by the Ethics Review Board of Xiangya Hospital. The informed consent was obtained from all patients.

The patients were followed up at outpatient every 3 months for 2 years after partial penectomy (or LBIL). The age, BMI, PL, treatment (partial penectomy or partial penectomy + LBIL), having a partner, psychological factors, and sexual function were selected as monitored parameters. Sexual function was assessed with the IIEF-15 before partial penectomy and at the 6 month after partial penectomy (or LBIL)^2^. Psychological factors were assessed with Zung’s Self-Rating Anxiety Scale (SAS) and Self-Rating Depression Scale (SDS) before partial penectomy and at the 6 month after partial penectomy (or LBIL)^3^,^4^.

SPSS17.0 software was applied for statistical analysis. We compared the demographic characteristics of partial penectomy patients with partial penectomy + LBIL patients using t-test and chisquare test. We compared the IIEF-15 scores of partial penectomy patients with partial penectomy + LBIL patients using t-test. We compared the preoperative IIEF-15, SAS and SDS scores with the postoperative scores using t-test. The level of statistical significance was set at p < 0.05. We used linear regression analyses to explore associations between each IIEF-15 term and age, BMI, PL, treatment, partner, SAS score, and SDS score. After first performing univariate regression analyses, variables with a p value < 0.15 were entered in the multivariate model, p values ≤ 0.5 were considered to reflect statistical significance.

## Result

Of 51 patients potentially eligible for the study, 8 were excluded: 4 patients treated with radiotherapy or chemotherapy after partial penectomy; 2 patients developed recurrent disease; 1 patient refused to complete questionnaires; 1 patient lost follow-up after partial penectomy. The remaining 43 patients fulfilled the inclusion and exclusion criteria for the study. The median age of patients was 56 years, the median BMI was 24 g/m^2^, and the median PL was 4 cm. Of the 43 patients, 34 patients having a partner, 35 patients were performed LBIL after partial penectomy.

Table 1 shows the mean preoperative and postoperative scores of IIEF-15, SAS, and SDS. The preoperative IIEF-15 scores were significantly different from the postoperative scores for each IIEF-15 domain. The preoperative SAS/SDS scores were also significantly different from the postoperative scores. According to the postoperative SAS/SDS scores, 25 patients (58%) were anxiety (standard scores ≥50) and 17 patients (39%) were depression(standard scores ≥53) after surgery. Table 2 shows the mean postoperative IIEF-15 domain scores for patients performed partial penectomy and partial penectomy + LBIL. There was no statistically significant difference between the two groups in demographic characteristics. The IIEF-15 scores of patients treated with partial penectomy were not significantly different from those treated with partial penectomy + LBIL.

### Erectile function

After partial penectomy, 21 (48.8%) patients reported erectile function that “most times” or “always” allowed sexual intercourse. 12 patients reported erection of the penile “sometimes” or “a few times” hard enough for penetration. 10 patients reported having “no sexual activity” or “almost never”. On multivariate analysis, age and SAS score were negatively associated with erectile function. (Table 3).

### Orgasmic function

When had sexual stimulation or intercourse after partial penectomy, 28 patients reported “most times” or “always” ejaculate or have the feeling of orgasm, 5 patients reported “sometimes” or “a few times” ejaculate or orgasm, 10 patients reported having “no intercourse”, or “almost never” ejaculate or orgasm. On multivariate analysis, SAS score was negatively associated with orgasmic function. (Table 3).

### Sexual desire

26 patients reported they “most times” or “always” had felt sexual desire after surgery and with a “high” or “very high” level of desire, 12 patients reported “sometimes” or “a few times” had felt sexual desire, 5 patients reported “almost never” had felt sexual desire. On multivariate analysis, age and SAS score were negatively associated with sexual desire. (Table 3).

### Intercourse satisfaction

12 patients reported they had more than seven attempts sexual intercourse one month after surgery. 19 patients reported they had three to six attempts sexual intercourse one month. 12 patients reported they had “one to two” or “not attempts” sexual intercourse. When attempted sexual intercourse, 16 patients “most times” or “always” felt satisfied, 16 patients “sometimes” or “a few times” felt satisfied, 11 patients “never” felt satisfied or “not attempt intercourse”. 10 patients reported their sexual intercourse “highly” or “very highly” enjoyable. 19 patients reported their sexual intercourse “fairly enjoyable”. 14 patients reported their sexual intercourse “no enjoyment” or they “no intercourse”. On multivariate analysis, SAS score was negatively associated with intercourse satisfaction; PL was positively associated with intercourse satisfaction. (Table 3).

### Overall satisfaction

7 patients reported they were “very or moderately satisfied” with their overall sex life or sexual relationship after surgery. 28 patients reported they were “about equally satisfied and dissatisfied” or “moderately dissatisfied”. 8 patients reported they were “very dissatisfied”. On multivariate analysis, age was negatively associated with overall satisfaction. (Table 3).

## Discussion

While penile carcinoma is a rare malignant disease, it has significant physiological and psychological effects on the patient. A variety of treatment modalities exist for patients with penile carcinoma, which dependent on the tumor location, size and stage. Aggressive therapy with partial penectomy is a common treatment for penile carcinoma patients. Although there is a reduction in the length of penis, several studies have reported that the remaining penis may still become erect and vaginal penetration is frequently possible^5^. In a Brazilian study, 64% of patients reported the overall sexual function was decreased after partial penectomy^6^. In another Brazilian study, 55.6% of patients reported erection of the penile allowed vaginal penetration, and all terms of sexual functioning reduced after partial penectomy^1^. However, the sample size of these studies were small, only 9 and 18 patients were included in each study. In our study, statistically significant reductions were observed after partial penectomy in each IIEF-15 term.

The psychological states were also getting worse. According to SAS/SDS scores after surgery, 25 patients (58%) were anxiety and 17 patients (39%) were depression. Few studies investigating psychological states after partial penectomy, and their results were controversial. In the study of Ficarra, the General Health Questionnaire showed impaired well being in 37.5% of patients after partial penectomy^7^. While in D’Ancona study no patients exhibited such impairment^6^. This variation is likely to be due to differing assessed methods of psychological factors and small sample size.

Few studies had evaluated the effect of inguinal lymphadenectomy on sexual function for penile carcinoma patients. Intraoperative nerve damage and postoperative lymphedema are both likely to affect sexual function. In Kieffer study, patients treated with lymphadenectomy were not significantly different from those without lymphadenectomy on sexual function^8^. However, those treated with lymphadenectomy reported more life interference. In our study, the IIEF-15 scores of patients treated with partial penectomy only were not significantly different from those treated with partial penectomy + LBIL. This may because the saphenous vein was preserved during lymphadenectomy in our study, which could reduced the morbidity of lymphedema^9^. We assessed the postoperative sexual function at the 6 month after inguinal lymphadenectomy, the potential nerve damage may be repaired during this period.

We found that the sexual function of penile carcinoma patients after partial penectomy was negatively affected by age and SAS score. As mentioned above, the morbidity of anxiety and depression were significantly increased after partial penectomy, more than half of the patients had experienced psychological problems. As psychological states were significantly related to sexual function, multidisciplinary follow up with psychologists is necessary.

Intercourse satisfaction was positively influenced by PL in our study. Longer penile length after partial penectomy was benefit for sexual function. The incision site is marked at 2 cm from the tumor in our study when performed partial penectomy. In Agrawa study, the specimens were evaluated histologically for grade and for proximal microscopic extensions beyond the visible penile tumor margin, by examining serial proximal 5 mm sections^10^. They found a 10 mm clearance is adequate for grade 1 and 2 lesions, and 15 mm for grade 3 tumors. This indicated that the incision site less than 2 cm from the tumor can be used for grade 1 and 2 patients.

Partner support is an important factor that helped patients to overcome the difficulties after partial penectomy^11^,^12^. The BMI of patients and depression in carcinoma patients are also two important factors that related to disease progression^1^. Having a partner was univariately positively associated with overall satisfaction, BMI was univariately negatively associated with intercourse satisfaction, SDS score was univariately negatively associated with erectile function, orgasmic function, sexual desire, intercourse satisfaction. However, they were not statistically significant associated with sexual function in the multivariate models. An explanation for this lack of statistical significance could be that our study sample size was small or some possibly confounding factors that not included in our multivariate model might influenced the result.

Although this is a relatively large sample size prospectively study compared to earlier studies, only 43 patients were included in our study. More subgroup analysis was not allowed, because the sample size was not sufficient. Another limitation was no control group was included in our study, which might induce selection bias. Further large-scale studies with control group should be performed to investigate the sexual function of penile carcinoma patients.

In conclusion, our preliminary findings suggest that the sexual function after partial penectomy was significantly reduced. The sexual function was negatively affected by age and anxiety but positively affected by PL.

## Additional Information

**How to cite this article**: Yu, C. *et al*. Sexual Function after Partial Penectomy: A Prospectively Study From China. *Sci. Rep*. **6**, 21862; doi: 10.1038/srep21862 (2016).

## Figures and Tables

**Table 1:** Preoperative and postoperative scores of IIEF-15, SAS, and SDS.

IIEF-15 scores	Preoperative	Postoperative	P
erectile function	26.70 ± 3.07	17.81 ± 10.66	<0.01
orgasmic function	8.44 ± 1.16	5.81 ± 3.35	<0.01
sexual desire	8.33 ± 1.27	6.28 ± 2.16	<0.01
intercourse satisfaction	12.30 ± 2.21	7.07 ± 4.56	<0.01
overall satisfaction	8.00 ± 1.19	5.91 ± 2.01	0.01
SAS scores*	46.30 ± 8.73	54.72 ± 11.74	0.01
SDS scores*	43.60 ± 8.32	51.26 ± 10.07	0.04

^*^Standard scores = original score multiply by 1.25.

**Table 2:** Mean postoperative IIEF-15 domain scores for patients underwent partial penectomy and partial penectomy + LBIL.

IIEF-15 scores	Partial penectomy n = 8	Partial penectomy + LBIL n = 35	P
erectile function	13.75 ± 10.17	18.74 ± 10.69	>0.05
orgasmic function	6.00 ± 3.55	5.77 ± 3.36	>0.05
sexual desire	5.25 ± 1.83	6.51 ± 2.19	>0.05
intercourse satisfaction	7.13 ± 4.99	7.06 ± 4.54	>0.05
overall satisfaction	6.13 ± 2.36	5.86 ± 1.96	>0.05

**Table 3:** Univariate and multivariate linear regression analyses per IIEF-15 domains.

Variables	Age		BMI		PL		Partner		Treatment		Postoperation SAS scores*		Postoperation SDS scores*	
IIEF-15 domains	Beta	P value	Beta	P value	Beta	P value	Beta	P value	Beta	P value	Beta	P value	Beta	P value
Erectile function														
Univariate	−0.46	<0.01			0.31	0.05					−0.56	<0.01	−0.32	0.04
Multivariate	−0.35	0.01									−0.57	<0.01		
Orgasmic function														
Univariate	−0.35	0.02			0.26	0.09					−0.53	<0.01	−0.30	0.05
Multivariate											−0.56	<0.01		
Sexual desire														
Univariate	−0.41	<0.01			0.24	0.12					−0.50	<0.01	−0.29	0.06
Multivariate	−0.31	0.04									−0.51	0.01		
Intercourse satisfaction														
Univariate	−0.35	0.03	−0.23	0.14	0.35	0.02					−0.46	<0.01	−0.23	0.13
Multivariate					0.27	0.05					−0.51	0.01		
Overall satisfaction														
Univariate	−0.42	<0.01			0.28	0.07	0.24	0.13			−0.51	<0.01	−0.42	<0.01
Multivariate	−0.29	0.05												

^*^Standard scores = original score multiply by 1.25.

## References

[b1] Romero F. R. . Sexual function after partial penectomy for penile cancer. Urology. 66, 1292–1295 (2005). 16360459 10.1016/j.urology.2005.06.081

[b2] Rosen R. C. . The international index of erectile function (IIEF): a multidimensional scale for assessment of erectile dysfunction. Urology. 49, 822–830 (1997). 9187685 10.1016/s0090-4295(97)00238-0

[b3] Zung W. W. A self-rating depression scale. Arch Gen Psychiatry. 12, 63–70 (1965). 14221692 10.1001/archpsyc.1965.01720310065008

[b4] Zung W. W. A rating instrument for anxiety disorders. Psychosomatics. 12, 371–379 (1971). 5172928 10.1016/S0033-3182(71)71479-0

[b5] Stoudemire A., Techman T. & Graham S. D. Jr. Sexual assessment of the urologic oncology patient. Psychosomatics. 26, 405–408, 410. (1985). 3991867 10.1016/S0033-3182(85)72848-4

[b6] D’Ancona C. A. . Quality of life after partial penectomy for penile carcinoma. Urology. 50, 593–596 (1997). 9338738 10.1016/s0090-4295(97)00309-9

[b7] Ficarra V. . General state of health and psychological well-being in patients after surgery for urological malignant neoplasms. Urol Int. 65, 130–134 (2000). 11054029 10.1159/000064857

[b8] Kieffer J. M. . Quality of life for patients treated for penile cancer. J Urol. 192, 1105–1110 (2014). 24747092 10.1016/j.juro.2014.04.014

[b9] Abbas S. & Seitz M. Systematic review and meta-analysis of the used surgical techniques to reduce leg lymphedema following radical inguinal nodes dissection. Surg Oncol. 20, 88–96 (2011). 20005090 10.1016/j.suronc.2009.11.003

[b10] Agrawal A., Pai D., Ananthakrishnan N., Smile S. R. & Ratnakar C. The histological extent of the local spread of carcinoma of the penis and its therapeutic implications. BJU Int. 85, 299–301 (2000). 10671885 10.1046/j.1464-410x.2000.00413.x

[b11] Witty K. . The impact of surgical treatment for penile cancer – patients’ perspectives. Eur J Oncol Nurs. 17, 661–667 (2013). 23896180 10.1016/j.ejon.2013.06.004

[b12] Bullen K., Edwards S., Marke V. & Matthews S. Looking past the obvious: experiences of altered masculinity in penile cancer. Psychooncology. 19, 933–940 (2010). 19862691 10.1002/pon.1642

